# Impact of heat stress on ruminant livestock production and meat quality, and strategies for amelioration

**DOI:** 10.1093/af/vfad046

**Published:** 2023-10-13

**Authors:** Surinder S Chauhan, Minghao Zhang, Richard Osei-Amponsah, Iain Clarke, Veerasamy Sejian, Robyn Warner, Frank R Dunshea

**Affiliations:** School of Agriculture, Food and Ecosystem Sciences, Faculty of Science, The University of Melbourne, Parkville, Melbourne, VIC 3010, Australia; Department of Food and Nutrition, Provincial Hospital, Shandong First Medical University, China; School of Agriculture, Food and Ecosystem Sciences, Faculty of Science, The University of Melbourne, Parkville, Melbourne, VIC 3010, Australia; Department of Animal Science, School of Agriculture, University of Ghana, Accra, Ghana; School of Agriculture, Food and Ecosystem Sciences, Faculty of Science, The University of Melbourne, Parkville, Melbourne, VIC 3010, Australia; Animal Physiology Division, ICAR-National Institute of Animal Nutrition and Physiology, Bangalore 560030, India; School of Agriculture, Food and Ecosystem Sciences, Faculty of Science, The University of Melbourne, Parkville, Melbourne, VIC 3010, Australia; School of Agriculture, Food and Ecosystem Sciences, Faculty of Science, The University of Melbourne, Parkville, Melbourne, VIC 3010, Australia; Faculty of Biological Sciences, The University of Leeds, Leeds LS2 9JT, UK

**Keywords:** adaptation, animal breeding, climate change, meat safety, thermotolerance

**Implications**

- Climate change will continue to accentuate the negative impact of heat stress (HS) on ruminant livestock production, compromising animal welfare and meat quality.
- Mitigation strategies, including providing shade on farms, modifications of animal housing (heat extractors, fans, water sprinklers, and cool drinking water), and nutritional interventions, are important short-term measures to reduce the negative effects of HS.
- Climate-smart breeding for thermotolerance and matching of adapted ruminant breeds to appropriate production systems should be considered for more sustainable livestock production systems.

## Introduction

**Figure 1.**
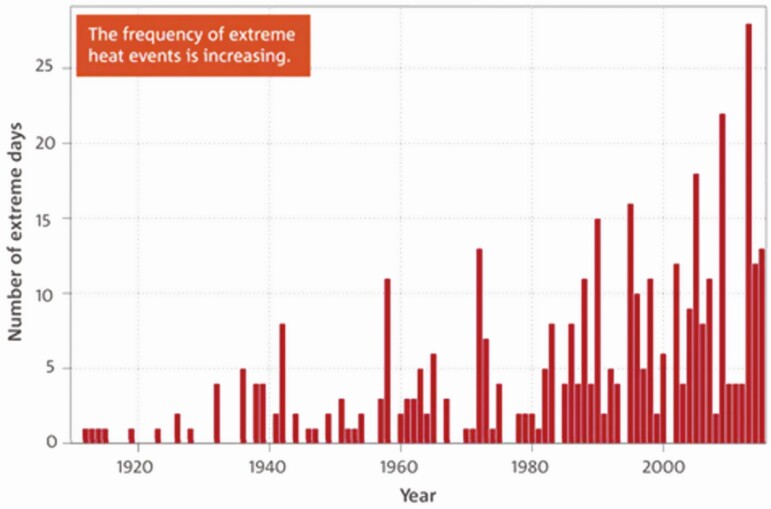
Frequency of extreme heat events in Australia. Source: Australian Bureau of Meteorology.

The Intergovernmental Panel on Climate Change (IPCC) projects a 1.5 °C to 2 °C increase in global warming in the 21st century, meaning that heat stress (HS) will continue to affect the sustainability of livestock farms worldwide negatively (Ciliberti et al., 2022), including reduced productivity of rangeland, shortage of nutritional feed, compromised animal welfare, and high energy costs for cooling (Ortiz-Colón et al., 2018). Additionally, heatwaves are becoming more frequent and of higher magnitude and are persisting for longer periods, compromising animal welfare and production in the absence of suitable interventions to counteract HS. For instance, the Australian Bureau of Meteorology (BOM) has recorded an annual increase in heatwaves in recent years (Figure 1), a trend that is expected to continue.

**Figure 2.**
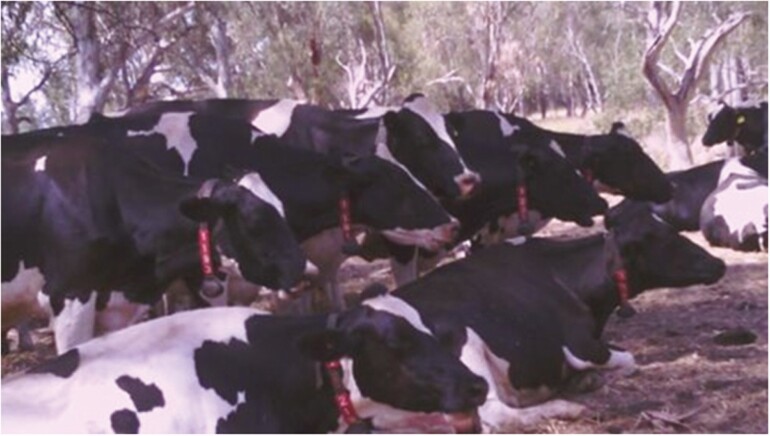
Dairy cattle at the University of Melbourne Dookie Robotic Dairy Farm resting under trees on a hot day during summer of 2018 (Osei-Amponsah et al., 2020).

Over millennia, ruminant livestock have developed behaviors to cope with high temperatures, through natural or artificial selection. For example, ruminants tend to rest during the hottest parts of the day and become more active during cooler times. Dairy cattle graze less during the hottest period of the day, seeking shade and spending more time at the watering points to conserve energy and reduce heat production (Figure 2).

## Direct and Indirect Effects of HS on Ruminant Production

Ruminant livestock are raised within diverse cultural and environmental production systems globally and contribute to food security. They have well-developed thermoregulatory mechanisms but adaptation to increased environmental temperature compromises animal performance production (Gaughan et al., 2019). Like other homeotherms, ruminants maintain their core body temperature within a narrow zone by maintaining a balance between heat gain and heat loss. Ruminants reduce their feed intake, production, and growth rate, to reduce metabolic heat production, a significant contributor to the total heat load on animals (Yadav et al., 2016; Sejian et al., 2017). To maximize radiant heat loss from the body, heat-stressed animals redistribute blood supply away from the gastrointestinal tract to the periphery resulting in hypoxia and cellular damage to intestinal membranes leading to leaky gut and inflammation, all of which can result in localized and general oxidative stress (OS) (Chauhan et al., 2014). The OS is an imbalance between oxidants and antioxidants at the cellular level or the entire organism, which damages macromolecules such as lipids and proteins and may result in reduced meat quality and product deterioration(Chauhan et al., 2014). HS in animals during the preslaughter period over summer reduces liveweight and carcass weight; causes pale, soft, and exudate (PSE), and dark, firm, and dry (DFD) meat in livestock; and increases the foodborne disease outbreaks, when compared to winter conditions (Gonzalez-Rivas et al., 2020). Similarly, exposure of pregnant animals to HS leads to significant adverse effects on their innate and adaptive immune functions and their offspring, influencing morbidity, mortality, and growth rate in heifers (Dahl et al., 2020).

**Figure 3.**
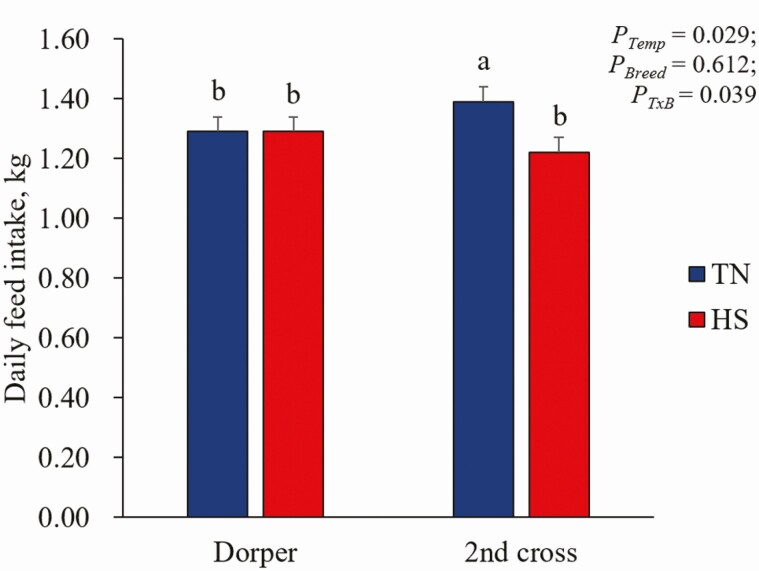
The effect of 2-wk HS or TNon feed intake of Poll Dorset × (Merino × Border Leicester; high production) and Dorper (high heat tolerance) lambs (n = 12 for each group). “a” and “b” indicate levels that are significantly different at 5% level of SED (Zhang et al., 2021). SED is shown as vertical bar.

HS also has indirect effects on ruminant production through a reduction in the quantity and quality of fodder while increasing the spread of new vector-borne diseases (Sejian et al., 2018). Generally, exposure to high ambient temperatures requires at least a week to influence sheep body weight. It takes 7 d of HS (28 °C to 40 °C cyclic HS) to negatively affect the bodyweight of lambs when compared to animals in a thermoneutral (TN: 18 °C to 21 °C) environment (Chauhan et al., 2016). For older sheep, this threshold may be longer due to conditioning to temperature change in previous seasons. Breed is another important determinant of heat tolerance in livestock, with hair sheep having thinner coats, more efficient sweating and shedding capacity, leading to superior heat tolerance, compared to sheep with wool (Sejian et al., 2018). Furthermore, high-production temperate breeds have poorer heat tolerance due to inherently higher metabolic rate and heat production than tropical breeds. Thus, we have shown that 2 wk of cyclic HS impacted Merino × Border Leicester lambs’ growth performance and feed intake but had no impact on Dorper lambs (Figure 3), which are adapted to hot climates.

## Effect of HS on Meat Quality of Climate-Adapted Ruminants

Climate-adapted animals have evolved physiological and behavioral mechanisms that allow the maintenance of body temperature within a narrow range, even when exposed to high temperatures. Climate-adapted phenotypes may be procured through either natural or artificial selection. Physiological mechanisms and behavioral changes conferring heat tolerance reduce the negative impact of HS on meat quality leading to reduced muscle fatigue which may improve meat tenderness. Additionally, changes in metabolism minimize the breakdown of fats, which can improve the flavor and aroma of the meat. However, severe, or prolonged HS can impact even climate-adapted animals. Under such conditions, the animal’s coping mechanisms may become overwhelmed, negatively impacting meat quality.

In general, reduction in meat production has been demonstrated in both long-term and short-term HS studies (Gregory, 2010; Thornton et al., 2022) (Table 1). Pragna et al. (2018a) found that the average body weight gain was lower in goats subjected to 45 d of summer conditions (73.5 to 86.5 Temperature Humidity Index [THI]) than in animals that were housed at a lower THI (69.9 to 74.9), confirming findings by Chauhan et al. (2016). Our current research indicates that less than 5 d of HS (28 °C to 40 °C, 30 % to 40% RH, cyclic temperature from day to night) does not affect body weight and daily weight gain of Merino crossbred lambs (Zhang et al., 2022). Therefore, the negative effects of HS on meat production (growth) do not manifest until at least 7 d of heat exposure with normal feeding, although feedstuffs with low antioxidant contents may aggravate this situation.

**Table 1.** The effect of heat stress on growth in small ruminants

Author	Breed	HS condition	Body weight change	Observation
Pragna et al. (2018b)	Osmanabadi goats Malabari goats Salem Black goats	Summer exposure: 73.5 to 86.5 THI Shed feeding: 69.9 to 74.9 THI 45-d study	ADG^1^:	HS significantly reduced the body weight gain among all heat exposed groups, but the reduction of feed intake of HS group was not significant (except for Malabari goats).
			Osmanabadi: Exposure −39.63^a^ g Shed 48.02^b^ g	
			Malabari: Exposure−25.00^a^ g Shed 39.29^b^ g	
			Salem Black: Exposure −21.03^a^ g Shed 34.53^b^ g	
Nicolás-López et al. (2021)	Dorper × Katahdin male lambs (34.6 ± 1.4 kg; 4.5 mo)	Summer: 28.3 ± 4.0 °C, 77.2 ± 5.4 THI Winter: 19.2 ± 2.6 °C, 64.0 ± 3.0 THI 30-d study	ADG:	The average body weight gain and feed efficiency of summer group were significantly lower than winter group.
			Dorper × Katahdin: Summer 226^a^ g Winter 302^b^ g	
Zhang et al. (2021)	Poll Dorset × (Border Leicester × Merino) lambs Dorper lambs	HS: 28 °C to 38 °C, 40% to 60% RH cyclic TN: 18 °C to 21 °C, 45% to 55% RH 2-wk study	ADG:	Two weeks of cyclic HS had a significant negative influence on feed intake and body weight gain of wool breed lambs (2nd cross), but the influence of HS was not significant for hair breed (Dorper lambs).
			Dorper: HS −50.6^a^ g TN 5.95^a^ g	
			2^nd^ cross: HS −92.3^a^ g TN 101.0^b^ g	
Chauhan et al. (2016)	White Suffolk × Merino × Border Leicester lambs (42 ± 2.0 kg; 7 mo)	HS: 28 °C to 40 °C, 30% to 40% RH TN: 18 °C to 21 °C, 40 % to 50% RH	Feed intake: HS 959^a^ g/d TN 1266^b^ g/d	One week of HS had a significant impact on 2nd cross lambs’ feed intake compared with the TN group.

^1^ADG, average body weight gain.

Carcass quality is affected by HS, as reflected in the quantity of subcutaneous and intramuscular fat (IMF). Limited research (Park et al. (2018) suggests that mild HS may be beneficial to fat deposition, conferring less subcutaneous fat and more IMF, but studies in small ruminants (Archana et al., 2018; Zhang et al., 2021) did not observe any impact of HS on subcutaneous fat thickness or IMF content. Similarly, a recent study in goat breeds indigenous to India (Devapriya et al., 2021) showed that, despite a higher magnitude of HS exposure (THI = 94.76) for a prolonged duration of 45 d, both the major carcass traits and meat quality variables remained unaffected. Although mild HS of less than 2 mo is unlikely to change the carcass fat content of ruminants, the effect of severe HS to reduce the subcutaneous fat thickness and IMF due to the loss of body weight requires further research.

**Figure 4.**
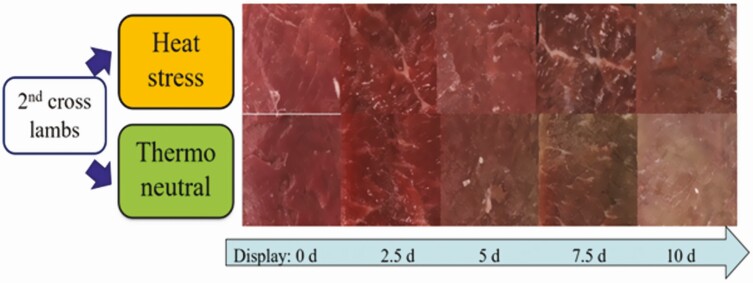
Effect of 2-wk HS or TN on meat retail display of Poll Dorset × (Merino × Border Leicester; high production) lambs’ *longissimus thoracis et lumborum* muscle in 10-d high-oxygen-modified atmosphere packaging (80% O_2_: 20% CO_2_; *n* = 12 for each group; Zhang et al., 2021).

Meat color, which depends on the concentration and chemical state of myoglobin, is influenced by a change in meat pH and oxidation state under HS conditions. Current data suggest that HS tends to increase the redness of meat (a*) and reduce lightness (L*) and color stability due to the higher ultimate pH and oxidation state, which causes meat to appear darker than normal (Zhang et al., 2020). It should be noted, however, that the increase of a* is not defined as “problem meat.” Thus, in a recent study, meat from lambs exposed to 2 wk of HS (2 wk; 28 °C to 40 °C, 40% to 60%) had better color stability (less browning; Figure 4) compared with meat from animals under TN conditions (Zhang et al., 2021). Therefore, unlike the seasonal impact of HS on meat quality, production and animal welfare outcomes are more likely to be compromised during the short-term high-ambient-temperature environment conditions.

**Figure 5.**
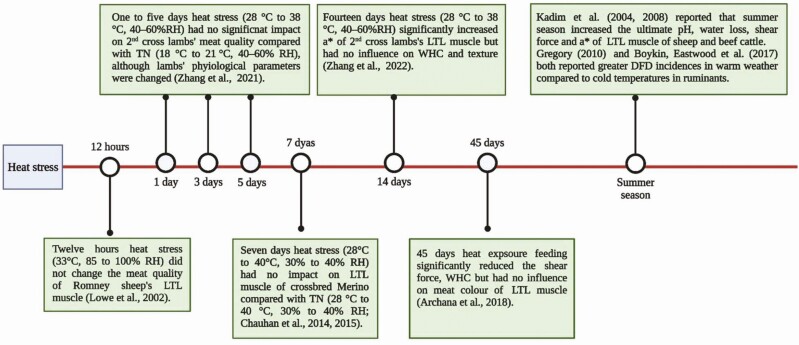
Summary of literature on effects of HS for different periods on meat quality in ruminants.

As indicated above, effects of HS are mitigated in climate-adapted animals. Our unpublished data show that wool-shedding breeds of sheep displayed higher meat color stability compared with wool breeds under cyclic HS conditions (Zhang et al., 2022). Compared to water holding capacity (WHC) and texture, meat color parameters are more sensitive to HS. For instance, in a recent study (Zhang et al., 2021), we showed that after 2-wk HS (28 °C to 38 °C, 40% to 60% RH; cyclic temperature) an increase in lamb meat redness (a*) becomes apparent but in other studies, the influence on WHC and texture was observed only after 1 mo of exposure to hot conditions as indicated in Figure 5 (Kadim et al., 2008; Archana et al., 2018).

One of the most consistent negative consequences of HS in ruminants is the increased frequency of DFD meat (Gregory, 2010) based on the higher meat ultimate pH. However, heat-stressed ruminants produce meat with lower WHC (Kadim et al., 2008; Archana et al., 2018), which is usually associated with lower ultimate pH and may be due to oxidative modification of proteins because of OS induced by HS. In addition to WHC, HS may also affect meat tenderness. For example, seasonal HS (35 °C, 47% RH) in sheep reduced the myofibrillar fragmentation index (MFI; lower MFI indicating less tender meat) of meat, compared with that of animals in the cool season (21 °C, 59%RH) (Kadim et al., 2008). Similarly, 45 d of HS exposure in goats increased shear force of meat as compared to meat produced from goats housed at lower THI (Archana et al., 2018). Macías-Cruz et al. (2020) confirmed these results, showing that Warner–Bratzler shear force of Dorper × Katahdin lambs’ *Longissimus thoracis* (LT) muscle from animals subjected to a month of summer conditions (28.4 ± 4.0 °C, 55.2 ± 18.2% RH) was higher than that of meat from animals subjected to winter conditions (19.2 ± 2.6 °C, 41.7 ± 11.0% RH). At least 2 wk of HS in ruminants is required before any impact on WHC and shear force of their meat occurs (Zhang et al., 2021). A summary of the literature on effects of HS on meat quality is given in Figure 5.

## How Does HS Change Meat Quality in Ruminants?

Until now, studies of the impact of HS on meat quality have focused mainly on the effect on postmortem muscle glycolysis. HS alters the physiology and metabolism of animals, including effects on redox balance and apoptosis. Thus, to develop suitable strategies to alleviate HS, it is important to consider how HS changes biological processes that affect meat quality. Lower glycogen concentrations due to reduced feed intake and hyperthermia under HS can predispose muscle to a higher ultimate pH, which is close to the definition of DFD meat (pH ≥ 5.8 or 6.0). If glycogen concentrations are above the threshold for postmortem glycolysis utilization, exposure to acute HS before slaughter may lead to a more rapid decline in muscle pH resulting in PSE meat (Gonzalez-Rivas et al., 2020). Generally, more than 2 wk of HS is required to elevate the ultimate pH of meat from sheep and beef cattle (Kadim et al., 2004; Rana et al., 2014; Zhang et al., 2021).

HS is also known to cause OS. In healthy biological systems, animals maintain a balance between reactive oxygen species (ROS) and antioxidants at the cellular level (Chauhan et al., 2014). However, HS leads to excessive ROS production and reduced antioxidants due to lower feed intake and increased requirements to scavenge ROS. For example, Shi et al. (2020) reported that 28 d of HS (25.25 °C, 74.49% RH) significantly reduced glutathione peroxidase activity and raised the malondialdehyde concentration of crossbred lambs (Dorper × Mongolian) compared with TN. Similarly, in a study of Merino crossbred lambs, we (Chauhan et al. (2016) observed that oxidation products in plasma were higher in lambs subjected to HS (28 °C to 40 °C, 30% to 40% RH) for 7 d than in lambs maintained under TN (18 °C to 21 °C, 40% to 50% RH) conditions, influencing the color stability of meat in the former. Moreover, high levels of ROS production during HS are likely to cause protein oxidation (such as myosin, sarcoplasmic proteins, calpain, myoglobin, and glycolytic enzymes) in meat leading to a change in muscle structure by cross-linking or denaturation, resulting in a reduction in the water space in muscle and decreased tenderness (Mitra et al., 2017). Therefore, further studies on antioxidant supplementation or the effects of adding natural herbs to the ruminant diet to reduce OS and improve meat quality are warranted.

## Effect of HS on Meat Safety

It is generally accepted that meat with high ultimate pH and intestinal tissue damage in live animals poses safety risks for meat and by-products. Some psychrotrophic bacteria (which grow at refrigeration temperatures and lead to the spoilage of product) are inhibited at normal pH (5.6) of meat but can grow on meat with a high ultimate pH, including *Acinetobacter* and *Altermonas putrefaciens* (Newton and Gill, 1981). On the other hand, tissue damage and inflammation of the intestines of ruminants induced by HS increases the intestinal permeability and luminal attachment of bacteria (Bailey et al., 2004). An increase in the levels of stress hormones such as catecholamine and glucocorticoids may also change intestinal barrier function and the microbial environment in ruminants (Verbrugghe et al., 2012). For example, HS impairs intestinal integrity and increases intestinal permeability to endotoxins and translocation of pathogenic bacteria into the bloodstream (*Salmonella*, *Escherichia coli*, and *Campylobacter*) into the bloodstream (Lian et al., 2020). Such intestinal bacteria may be taken up by muscle and liver (dependent on the health of the animal), which can lead to foodborne illness in humans. Thus, HS can compromise meat safety, especially in low- to medium-input production systems in the developing world where ruminant production significantly contributes to food security.

## Strategies for the Amelioration of HS

Climate change induced HS, and its challenge to livestock production requires adaptation and mitigation strategies. There are some adaptive practices that have been developed in breeding and pasture management programs including selection for more heat-resistant genotypes (Gaughan et al., 2019), silvopasturing (the practice of integrating trees, forage, and the grazing of domesticated animals in a mutually beneficial way), and diversification of forage production and optimization production practices to reduce HS (Ortiz-Colón et al., 2018). Another strategy is to increase the energy and nutrient densities (reduced fiber, increased concentrates, and supplemental fat) of the diet since feed intake is reduced under HS. Increasing ration concentrates should be considered with care; however, as heat-stressed cows, for instance, are highly prone to rumen acidosis (Baumgard et al., 2014). The risk of increased concentrate feeding can be mitigated by feeding slowly fermentable grains such as maize or by treatment of rapidly fermentable grains to reduce rumen starch fermentation (Gonzalez-Rivas et al., 2016, 2017, 2021; Prathap et al., 2022). Another strategy to mitigate the effects of HS in ruminant livestock is the physical modification of animal’s environment to reduce direct exposure to heat ranging from the provision of shade, and cooling animals down using sprinklers, fans, mists, and perforated air ducting systems (Gaughan et al., 2019). Nutritional interventions to protect against oxidative damage resulting from HS-induced ROS production and antioxidant supplementation with betaine, lipoic acid, selenium, Vitamins C and E can also be utilized in combination with physical modification of the environment (Osei-Amponsah et al., 2019). However, these strategies often depend on the availability of electrical energy, adequate water supply, availability and affordability of nutritional supplements, and more capital investment often not available in all production systems.

A key component of adaptation is the innate genetic ability of an organism to survive under stressful conditions (Osei-Amponsah et al., 2019). Climate-adapted animals (Pereira et al., 2014; Cardoso et al., 2015) have evolved specific physiological and behavioral mechanisms that help regulate their body temperature to maintain homeostasis. For example, sweating allows the animals to release heat from their bodies through evaporation, while panting increases airflow over the respiratory system, allowing heat dissipation. Animals reduce their metabolic activity and heat production to cope with HS (Gonzalez-Rivas et al., 2020). In cattle, thermo-tolerance is directly associated with sweat gland density and sweating rate. Cattle adapted to arid regions possess smooth, short, and thin hair (slick hair gene) coat, which enhances heat dissipation from the body (Dikmen et al., 2018). Sheep with light coat colors, which are sleek and shiny, reflect greater solar radiation than hair coats that are dark and dense or woolly (Sejian et al., 2018). Furthermore, sheep with carpet wool are better protected from solar radiation by the facilitation of cutaneous heat dissipation (Sejian et al., 2018). Sheep with longer, thicker, and darker coats are more substantially stressed and exhibit higher rectal temperatures and sweating rates in tropical regions than white-haired sheep (Gaughan et al., 2019). Goats are proficient desert-dwelling animals as their physiological characteristics provide them with an advantage over other ruminants in harsh environmental conditions. Their small body size, short pointed erect ears, fleece structure, light coat, and high digestive efficiency help them survive extreme climatic conditions (Silanikove, 1997).

Genetic selection for heat tolerance could provide a sustainable means of augmenting feeding and housing modifications. For instance, selection signatures for thermotolerance can be identified through functional genomics, and productive breeds improved through crossbreeding with resilient genotypes and the incorporation of stress-tolerant genes. An example is the SLICK haplotype (https://omia.org/OMIA001372/9913) initially identified in Senepol cattle and other South America breeds, which has been introgressed to Holsteins to improve their thermotolerance (Dikmen et al., 2008). Inheritance of the SLICK1 allele of the prolactin receptor gene improves the thermotolerance of lactating Holstein cows under hot-humid environmental conditions (Carmickle et al., 2022). Furthermore, local breeds, particularly those from the Near East and Africa, are known for their better adaptation to high temperatures and harsh conditions and generally have a greater adaptive capacity to stressful environments than exotic breeds (Osei-Amponsah et al., 2019). Therefore, breeding for thermotolerance and matching adapted ruminant breeds to suitable environments and production systems are useful strategies.

A combination of strategies (Baumgard et al., 2014; Sejian et al., 2018; Collier et al., 2019; Gaughan et al., 2019) will be needed to mitigate HS in ruminants:

- Provision of shade—Access to shaded areas can reduce direct exposure to sunlight and the animal’s body temperature. In the extensive dairy systems, shade from trees can form part of a long-term strategy for managing climate variability on farms. The shade and shelter provided by trees in paddocks and laneways can reduce the radiant heat load by 50% or more.
- Provision of adequate water to maintain hydration during hot weather.
- Adjusting feeding patterns—Feeding animals during cooler times, such as early morning or late evening, to reduce HS by lowering the heat produced during digestion.
- Adjusting feed composition—Feeding diets with a lower proportion of fiber and a higher proportion of more readily digestible carbohydrates can reduce heat production during digestion.
- Sprinklers or misting fans—Sprinkling or misting animals with water can help to cool them down and reduce HS.
- Improving ventilation—Adequate ventilation in housing can help to reduce heat build-up and improve air quality.
- Convective cooling—Fans can circulate air and reduce heat load on animals, which can help lower the core body temperature of animals.
- Provision of cool, dry bedding.
- Nutritional supplementation—In the short term, antioxidant supplements can reduce OS and betaine can reduce energy requirements.
- Genetic Interventions—In the longer term, selection for heat tolerance is an effective long-term strategy, as is introgression of specific (*SLICK*) genes. The application of OMICS technologies offers considerable scope for identification of more reliable biomarkers for HS resilience which could be incorporated in marker-assisted selection (MAS) to redefine the existing breeding goals for thermotolerance.

## Conclusions

Ruminant livestock are important contributors to global food production and an important income source for many farmers in developing countries. HS remains a major threat to ruminant livestock production, reducing feed intake, LWG, limiting meat and milk production, damaging the intestinal wall, compromising nutrient digestion and absorption, and affecting meat quality. Unfortunately, these harmful consequences of HS in terms of animal health and production are likely to be an ongoing issue across the globe, and a multidisciplinary approach integrating animal breeding, nutrition, and housing is required to counteract the impact of climate change on livestock. Amelioration strategies such as provision of shade, sprinklers, fans, energy-efficient cooling systems, water provision, feeding changes, and nutritional interventions can be utilized in the short term. However, it is important to note that some of these strategies may not be applicable to extensive systems and most of these require the use of electricity and water, which might not be a sustainable option in certain regions. In extensive systems, crossbreeding high-producing exotic breeds with local climate-adapted breeds, planting more trees in paddocks and laneways, and encouraging early morning and late afternoon grazing could be more useful.

In the short term, modification of the animal’s environment and nutritional interventions can be considered mitigation strategies to sustain livestock production in a changing climate. In the long term, genetic selection for thermotolerance and climate adaptation must be prioritized in future ruminant livestock breeding programs to improve animal comfort and performance in a changing climate. The integration of new technologies and capacity building in less-developed countries offers many opportunities for further development of strategies to reduce the negative impact of climate change on livestock.

*Conflict of interest statement*. None declared.

## About the Author(s)

**Surinder Singh Chauhan** is a Senior Lecturer in Animal Science, School of Agriculture, Food and Ecosystem Sciences (SAFES), The University of Melbourne, Australia. Dr. Chauhan’s research program strives to deliver solutions to promote sustainable and efficient livestock production systems capable of producing quality animal protein for consumers. Dr. Chauhan obtained his Bachelor and Master of Veterinary Science (Physiology) from HP Agriculture University Palampur, India, followed, PhD in Animal Sciences at The University of Melbourne and Postdoctoral in Muscle Biology and Meat Science at The Ohio State University, Columbus, USA. Dr. Chauhan is leading Australia–India collaboration for sustainable livestock production. His current research is focused on understanding the impact of climate change on farm animals and developing economic strategies to reduce the negative effects of heat stress on farm animal production and product quality. Surinder has many publications, has presented papers, and has been keynote speaker at international conferences.

**Minghao Zhang** is a Research Fellow in the Department of Food and Nutrition at the Provincial Hospital Affiliated with Shandong First Medical University. His main research focus is on the influence of climate change on meat quality and human health. Michael obtained his PhD at School of Agriculture, Food and Ecosystem Sciences (SAFES), Faculty of Science, University of Melbourne, Australia, on comprehensive assessments of the effects of different ambient temperatures and heat exposure durations on sheep and chicken meat quality. He was also responsible for a research project on the relationship between climate change and body temperature management in patients with diabetes.

**Richard Osei-Amponsah** is a William Gamble Visiting Fellow at The University of Melbourne Dookie Campus, School of Agriculture, Food and Ecosystem Sciences (SAFES), Faculty of Science, The University of Melbourne, Australia and an Associate Professor of Animal Science, University of Ghana. He holds a Doctor of Philosophy (PhD) degree in Animal Science (Animal Breeding and Genetics) from the University of Ghana, Legon. His research focuses on sustainable improvement and management of animal genetic resources and their adaptation to their production and changing environments. He has contributed to the development of sustainable animal breeding programs for low input-livestock systems, served as resource person at various stakeholder training workshops, supervised many students, and participated in several international and local conferences. Richard is an animal genetics consultant, has many publications to his credit, is a member of the All-Africa Society of Animal Production (AASAP), and is the current President of the Ghana Society of Animal Production (GSAP).

**Iain Clarke** has been a Research Professor in the School of Agriculture, Food and Ecosystem Sciences, The University of Melbourne since 2018. Prior to this, he held a Fellowship from National Health and Medical Research (Australia) as an independent scientist at Prince Henry’s Institute of Medical Research and then moved to Monash University, rising to the position of Head of Department of Physiology (2017–2017). He has graduated 18 PhD students. His original research was in the field of the neuroendocrinology of reproduction and other areas of neuroendocrinology (stress, metabolism). Later, he developed a strong interest in the neuroendocrinology of obesity. He has published several peer-reviewed papers/chapters/reviews and has received several national and international awards. His current research interest is in the field of animal science, with a special emphasis on the identification of thermotolerant animals. He is strongly committed to producing scientific data that may be translated into on-farm benefits.

**Veerasamy Sejian**, MVSc, PhD, is the Dean, Rajiv Gandhi Institute of Veterinary Education and Research (RIVER), Puducherry, India. Dr. Sejian is the team leader who established the concept of “Multiple stresses impacting small ruminant production in the changing climate scenario.” His current research is focused on identifying molecular markers for different environmental stresses in small ruminants with an objective of developing agro-ecological zone specific thermo-tolerant breeds. He has been the leader for an Indo-French collaborative project “Emerging Crop-Livestock Production Systems adapted to a Changing Environment (ECLIPSE Project).” Dr. Sejian has published three international Springer books on collaborative mode “Environmental stress and amelioration in livestock production”; “Climate change impact on livestock: adaptation and mitigation” and “Sheep production adapting to climate change.” For his outstanding contribution in climate change and livestock production, Indian Council of Agricultural Research (ICAR) has bestowed him with the prestigious Lal Bahadur Shastri Outstanding Young Scientist Award.

**Robyn Warner** is a Professor in Meat Science, Faculty of Science, The University of Melbourne, Australia. Her research uses fundamental muscle biochemistry and structure to investigate issues of importance to the meat industry. Her focus is on integrating novel techniques in biometrics and sensory to deeply understand consumer response to meat; developing capability and equipment to quantitate the flavor fingerprint of red meat in order to leverage consumer demand for unique experiences; understanding the effects of heat stress and climate change on meat quality; and developing fundamental understanding of how muscle proteins change during animal growth, nutrition, preslaughter stress, postmortem biochemistry, packaging, processing, and cooking. She is actively driving engagement with industry and serves on Meat Standards Australia assuring quality to Australian consumers of red meat and has won national and international prizes for this role. Robyn has many publications and has undertaken research with impact.

**Frank Dunshea** is a Redmond Barry Distinguished Professor and Chair of Agriculture at the University of Melbourne and Professor of Animal Growth and Development at the University of Leeds. He has a BAg Sci (Hons) and PhD from La Trobe University and postdoctoral training at Cornell University. He has more than 35 yr of research in growth physiology and nutrition and the use of domestic animals in nutritional and biomedical research. His research has had a high scientific impact, with many of the results being rapidly adopted by industry. Professor Dunshea is a respected research leader in global livestock industries and is committed to ensuring that livestock industries operate in a responsible and sustainable manner. In addition to many awards, he is a Fellow of the Australian Nutrition Society, the Australasian Pig Science Association, the Australian Society of Animal Sciences, and the International Union of Nutritional Sciences.
